# On-Demand Release of Protective Agents Triggered by Environmental Stimuli

**DOI:** 10.3389/fchem.2020.00304

**Published:** 2020-04-29

**Authors:** Chiara Giuliani, Elena Messina, Maria Paola Staccioli, Marianna Pascucci, Cristina Riccucci, Leonarda Francesca Liotta, Luca Tortora, Gabriel Maria Ingo, Gabriella Di Carlo

**Affiliations:** ^1^Institute for the Study of Nanostructured Materials (ISMN), National Research Council (CNR), Rome, Italy; ^2^Institute for the Study of Nanostructured Materials (ISMN), National Research Council (CNR), Palermo, Italy; ^3^Surface Analysis Laboratory, Istituto Nazionale di Fisica Nucleare (INFN) - Sezione di Roma Tre, Rome, Italy; ^4^Department of Sciences, Roma Tre University, Rome, Italy

**Keywords:** nanocarriers, inhibitors, corrosion, stimuli responsive, steel

## Abstract

The aim of this study was to develop smart materials with stimuli-responsive properties for the long-term protection of steel. The idea was to obtain a tailored and controlled release of protective agents in response to the environment stimuli. First, the protective efficacy of three inhibitors containing a carboxylic moiety, such as *p*-aminobenzoic (pAB), succinic (SA), and caffeic (CA) acids, was investigated in alkaline chloride solutions. The results revealed that pAB is the most effective protective agent, significantly better than SA and CA. It is surprising that the steel surface in the pAB solution remains unchanged even after 5 months of corrosion treatment, whereas the formation of degradation products in the SA and CA solutions was observed after only 6 days. Based on these findings, pAB was selected and used for the functionalization of silica nanoparticles and layered double hydroxides (LDHs) that can act as delivery vehicles and as an inhibitor reservoir. Specifically, pAB was chemisorbed on silica amino groups *via* an amide bond, and this makes possible a gradual inhibitor release induced by an alkaline environment. The intercalation of pAB in its anionic form into the LDHs structure is responsible for a completely different behavior since the release is induced by chloride ions and occurs by an anionic exchange reaction. Thus, these materials play a dual role by acting as an inhibitor reservoir and by capturing chlorides. These findings reveal that it is possible to create a reservoir of corrosion inhibitors gradually released on demand based on the chemical environment. The stimuli-responsive properties and the complementary protective action of inhibitor-loaded silica and LDHs make them attractive for the long-term protection of steel and open the way for innovative solutions in the preservation of concrete cultural heritage.

## Introduction

Chloride-induced corrosion of reinforcing steel is one of the major causes of degradation in concrete structures. During the past decades, the search for new protective materials has received considerable attention in order to hinder the degradation processes which can be responsible for irreversible damages (Bertolini et al., 2004; Goyal et al., 2018). The inhibition of steel corrosion is mandatory to increasing the service life of reinforced concrete structures and is particularly relevant to avoiding the loss of reinforced concrete monuments that are recognized as part of a cultural heritage. To face these issues, an attractive strategy is to develop novel solutions that can provide a long-lasting protection by considering safety and aesthetic aspects. The latter are necessary especially in cultural heritage applications.

Several approaches have been explored to prevent or slow down the corrosion processes of metals and their alloys (Bertolini et al., 2004; Rani and Basu, 2012; Yang et al., 2013; Saremi and Yeganeh, 2014; Giuliani et al., 2018; Goyal et al., 2018). Among them, the use of corrosion inhibitors is one of the most attractive since it can offer a simple and cost-effective prevention technique (Ormellese et al., 2009; Yang et al., 2016). In the last decades, organic inhibitors mainly based on mixtures of alkanolamines, amines, organic acids, or amino acids have received great interest since they represent an effective and safe alternative to common inorganic inhibitors (Jamil et al., 2003; Söylev and Richardson, 2008; Gartner et al., 2016). However, their efficiency may be reduced by certain factors, such as the evaporation of the volatile components (Elsener et al., 1999; Tritthart, 2003). Moreover, due to increasing awareness of the health and ecological risks, the search for environment-friendly inhibitors for steel represents an open issue. Further efforts are necessary to explore new interesting compounds, improve the efficacy of protective agents, and develop systems able to provide long-term action against corrosion.

A promising approach is to develop smart materials that can act as a reservoir of inhibitors and release them under external stimuli, thus prolonging their protective action. Due to their versatility, silica and layered double hydroxides (LDHs) have great potential to be used for the storage and controlled release of corrosion inhibitors (Yang et al., 2013, 2016; Saremi and Yeganeh, 2014; Rahsepar et al., 2017; Salzano de Luna et al., 2018). According to the literature, silica nanocontainers were studied, for example, as a host of molybdate ions or a mercaptobenzothiazole corrosion inhibitor (Saremi and Yeganeh, 2014; Rahsepar et al., 2017). Layered double hydroxides were intercalated with different inhibitor molecules, including organic acids (Yang et al., 2016). Several substances of potential interest have also been investigated for the corrosion inhibition of steel, among them organic acids and their derivatives, such as *p*-aminobenzoic (pAB), succinic (SA), and caffeic (CA) acids (Giacomelli et al., 2004; Amin et al., 2007; de Souza and Spinelli, 2009; Yang et al., 2016).

The novelty of this study is in selecting an environment-friendly organic corrosion inhibitor containing at least a carboxylic moiety and using the same molecule for the functionalization of both silica and the LDH nanocarrier. Moreover, the stimuli-responsive properties of the inhibitor-loaded nanocarriers were investigated in alkaline chloride solutions. To the best of our knowledge, a direct comparison between silica and the LDH nanocarriers loaded with the same inhibitor has not been reported so far.

The presence of a carboxylic group was preferred since it is suitable for chemisorption on silica and for the intercalation into the LDH structures. To select the protective agent, three corrosion inhibitors, pAB, SA, and CA, were compared and their protective efficacy was investigated in alkaline chloride solutions. The alkaline pH simulates the typical environment of steel reinforcement in concrete structures, whereas chloride ions promote the corrosion processes, thus assessing the protective efficacy in a short time. The best-performing candidate, pAB, was chemisorbed into silica nanoparticles and intercalated into a layered double hydroxide. The challenge was to develop stimuli-responsive materials able to release the inhibitor under pH variations and in the presence of chloride ions. In particular, pAB chemisorption on silica *via* amide bonds is aimed at a pH-dependent release, whereas the intercalation into LDHs is of interest for a chloride-induced release. Silica nanoparticles and LDHs functionalized with pAB were characterized by using complementary techniques and their stimuli-responsive properties were explored in the alkaline chloride solutions.

## Materials and Methods

### Materials

Silica nanoparticles with an average diameter of 5–15 nm and specific surface area of 540 m^2^/g, 3-aminopropyl)-triethoxysilane (APTES), 1-(3-(dimethylamino)propyl)-3ethylcarbodiimide (EDC), *N*-hydroxysuccinimide (NHS), *p*-aminobenzoic acid, 4-morpholineethanesulfonic acid (MES), ninhydrin, ethanol, and toluene were purchased from Sigma-Aldrich. Magnesium nitrate hexahydrate, aluminum nitrate non-ahydrate, and succinic acid were purchased from Carlo Erba.

### Preparation of Silica and LDHs With Inhibitor

#### Silica Nanoparticles With Inhibitor

##### Preparation of amino-functionalized silica nanoparticles (SiO_2_-NH_2_)

Amino-functionalized silica nanoparticles (SiO_2_-NH_2_) were prepared by dissolving 0.5 g of commercial silica nanoparticles (SiO_2_-NPs-15) in 30 ml of anhydrous toluene. Then, 1.3 ml of APTES was slowly added to the silica suspension under vigorous stirring. The resulting solution was refluxed at 120°C under stirring for 24 h. The nanoparticles were separated by centrifugation (10,000 rpm, 10 min; Thermo Scientific IEC CL31R Multispeed Centrifuge) and then purified by five cycles of centrifugation–dispersion in ethanol (50 ml), followed by vacuum drying at 60°C for 12 h.

##### Preparation of p-aminobenzoic-functionalized silica nanoparticles (SiO_2_-NH-pAB)

The carboxylic groups of pAB were activated with EDC in the presence of NHS. The use of water-soluble EDC and NHS avoids the use of organic toxic solvents in the synthesis. EDC (150 mg) and NHS (111 mg) were added to a solution of pAB (133 mg) in 8 ml of MES buffer (0.1 M, pH 5.5). The reaction was allowed to proceed under stirring at room temperature for 1 h. Subsequently, a suspension of 400 mg of amino-functionalized silica (SiO_2_-NH_2_) in 20 ml of MES buffer was added to the pAB solution. The mixture was kept in the dark, at room temperature, under stirring for 24 h. The obtained nanoparticles were separated by centrifugation (10,000 rpm, 10 min; Thermo Scientific IEC CL31R Multispeed Centrifuge), purified by repeated cycles of centrifugation and dispersion in water (two cycles) and ethanol (four cycles), and dried in air at room temperature for 24 h.

#### Layered Double Hydroxides With Inhibitor

MgAl-pAB was prepared by a pH-controlled co-precipitation synthetic method (Meyn et al., 1990; Aisawa et al., 2001; Kuang et al., 2010), which is the most common preparative technology for LDHs. This method is based on the hydrolysis of M^2+^ and M^3+^ hydroxide ions in the presence of pAB (Wang and O'Hare, 2012).

The Mg/Al ratio chosen for the synthesis was 1:1 to obtain stable layered compounds. Typically, 200 ml of a mixed solution of 0.25 M Mg(NO_3_)_2_·6H_2_O and 0.25 M Al(NO_3_)_2_·9H_2_O was slowly added dropwise to 200 ml of 0.125 M of the pAB solution at room temperature, with stirring under a nitrogen atmosphere to avoid contamination by atmospheric CO_2_ (He et al., 2004). The free organic acid was previously dissolved in a freshly prepared solution of NaOH (0.4 M) and the resulting solution stirred for 1 h under nitrogen.

The solution pH of the moisture remained fairly constant at 10 by dropwise addition of 1 M NaOH. The slurry was aged at 65°C for 24 h and then the resulting precipitate was washed extensively with water, centrifuged, and dried at 70°C under vacuum for a further 24 h. The product was labeled as LDH-pAB.

Mg–Al–MHT–NO_3_ was also synthesized by using the co-precipitation method, where an aqueous solution containing M^2+^ and M^3+^ ions was slowly added to a basic aqueous solution (NaOH). The precipitate was then filtered in a vacuum process and washed with deionized water in order to remove the excess nitrate. The solid was dried at 100°C in an oven for 12 h. The so-obtained material was labeled as LDH-NO_3_ and used as a comparison during the characterizations.

### Characterization

#### X-Ray Powder Diffraction

The measurements were performed in a Siemens 5000 X-ray powder diffractometer with a CuKα radiation (λ = 1.5418 Å) filtered by a nickel window. Angular values with a step size of 0.05° and a sampling time of 2 s were the experimental parameters used for data acquisition. In order to identify the crystalline phases, analysis of the X-ray diffraction patterns was carried out by using electronic databases.

#### FTIR Spectroscopy

The spectra were recorded using a Nicolet iS50 (Thermo Fisher) spectrometer equipped with an attenuated total reflectance (ATR) accessory. The measurements were recorded using a diamond crystal cell ATR using typically 32 scans at a resolution of 4 cm^−1^. No ATR correction has been applied to the data. The samples were investigated under the same mechanical force pushing the samples in contact with the diamond crystal.

#### Scanning Electron Microscopy

Morphological characterizations were performed by a high-brilliance and high-spatial-resolution LEO Gemini 1530 (Zeiss, Germany) field-emission scanning electron microscope (FE-SEM) equipped with an INCA 450 energy-dispersive X-ray spectrometer (EDS) and a four-sector backscattered electron detector (BSD).

#### Analysis of Amino Groups on Silica

The amino groups grafted on the silica nanoparticle surface were qualitatively detected and quantitatively determined by using the ninhydrin assay. Initially, ninhydrin in ethanol (2 ml, 25 mM) was reacted with APTES in ethanol at various known concentrations (0.2–0.85 mM). Ruhemann's Blue, with its characteristic intense absorption band at 584 nm, was formed by the selective reaction of ninhydrin with the primary amine groups. The absorbance at 584 nm of the above samples was recorded to establish the calibration curve by plotting the absorbance vs. the molarity of APTES. Thereafter, 10 mg of SiO_2_-NH_2_ was added to 2 ml of the ninhydrin ethanol solution (25 mM) and the mixture was heated at 90°C for 15 min. After cooling and centrifugation (10,000 rpm, 10 min; Thermo Scientific IEC CL31R Multispeed Centrifuge), the absorbance of the supernatant was measured at 584 nm against a blank reference (SiO_2_-NH_2_) by using a UV–Vis double-beam spectrophotometer (JASCO V 660). The supernatant was diluted in order to maintain the absorbance in the range 0.1–1. The concentration of the amine groups of SiO_2_-NH_2_ was determined by using the calibration curve of APTES.

#### TGA Analysis

SiO_2_-NH_2_ and SiO_2_-NH-pAB were characterized by thermogravimetric analyses (TGA) to evaluate the organic content of the surface-modified silica nanoparticles. SiO_2_-NPs-15 was used as a reference. TGA characterization was performed with a TGA/DSC1 STAR system from Mettler Toledo. The sample (15 mg) was treated under airflow (30 ml min^−1^) heating from 25 to 1,100°C (ramp rate, 10°C min^−1^).

### Corrosion Tests in Simulated Concrete Pore Solutions

Steel disks were immersed in a simulated concrete pore solution prepared by dissolving 0.001 wt% Ca(OH)_2_ in deionized water and fixing the pH at 10.5 using KOH (Gartner et al., 2016). In order to study the anti-corrosion performance of the three inhibitors selected (pAB, SA, and CA), an amount of 0.01 wt% of chloride ions was added to the solutions with a determined amount of inhibitor so as to reach a concentration of 50 mM. The disks were left in the simulated concrete pore solutions, and they were analyzed by means of optical microscopy after 40 h, 6 days, 20 days, and 5 months of immersion. The steel surface was also characterized by ATR-Fourier transform infrared (FTIR) spectroscopy after 6 days of corrosion tests.

### Tests of Inhibitor Release

#### *p*-Aminobenzoic Acid Release Experiments

The inhibitor release was investigated in alkaline solutions at a pH of 12.65, which represents the typical value for concrete pore solutions (Ormellese et al., 2006; Garcés et al., 2011). Initially, alkaline solutions (pH 12.65) of pAB at various known concentrations (1.75–4.25 × 10^−5^ M) were prepared and their absorbance was recorded. The absorbance of the above samples at 266 nm was plotted vs. the molarity of pAB to establish the calibration curve of pAB at pH 12.65. Thereafter, pAB-functionalized silica nanoparticles (20 mg) were placed in an Eppendorf tube containing 1.5 ml of aqueous solution at neutral and alkaline pH (pH 12.65). The suspensions were kept in the dark at 30°C under stirring for 552 h (23 days). At predetermined intervals of time, i.e., 3, 24, 48, 144, and 552 h, the mixtures were centrifuged for 10 min at 10,000 rpm (Thermo Scientific IEC CL31R Multispeed Centrifuge) and the collected supernatants were analyzed by UV–Vis spectroscopy (JASCO V 660 UV–Vis double-beam spectrophotometer). An alkaline solution (pH 12.65) of pAB was used as the control. After that, the original nanoparticles were redispersed with the corresponding supernatant. The molar concentration (in moles per liter) of the released pAB was evaluated as a function of time by the absorption at 266 nm using a calibration curve for pAB.

The stimuli-responsive properties of LDH-pAB were investigated by using a similar procedure and by monitoring the inhibitor release in solutions at neutral pH, at alkaline pH, and at alkaline pH in the presence of chloride ions. The amount of pAB released into the solutions was measured at different times by UV–Vis spectroscopy considering the absorbance at 266 nm.

## Results and Discussion

### Selection of Protective Molecules

**Figure 1:**
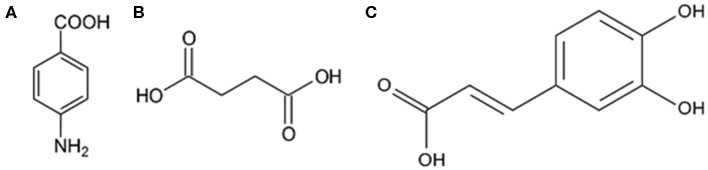
Structural formula of **(A)** *p*-aminobenzoic acid (*pAB*), **(B)** succinic acid (*SA*), and **(C)** caffeic acid (*CA*).

In order to select the protective molecule to be confined into stimuli-responsive carriers, three corrosion inhibitors were compared, namely, pAB, SA, and CA. All these molecules contain at least a carboxylic group, but they differ for the other functional groups and their steric hindrance, as shown in Figure 1, and this can have an effect on the protective performance. These inhibitors were identified based on literature data since they were recognized as effective in corrosion inhibition (Giacomelli et al., 2004; de Souza and Spinelli, 2009; Yang et al., 2016). However, the tests assessing inhibitor efficacy are usually carried out in experimental conditions that are sometimes difficult to compare. Therefore, in this study, pAB, SA, and CA were tested by using the same procedure.

**Figure 2:**
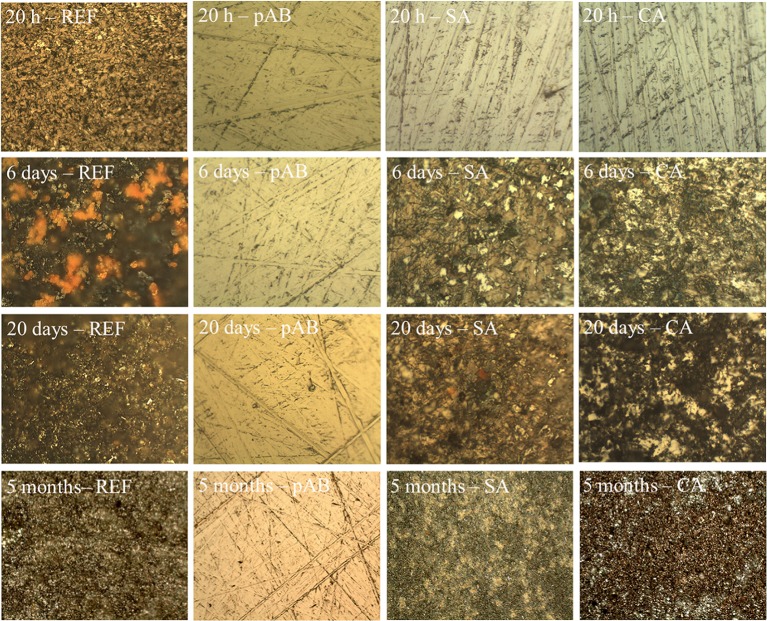
Optical micrographs of the steel surface after corrosion treatment for 20 h, 6 days, 20 days, and 5 months in simulated concrete pore solutions containing chloride ions. The tests were conducted without inhibitor (*REF*) and with *p*-aminobenzoic acid (pAB), succinic acid (SA), and caffeic acid (CA).

The protective efficacy of these inhibitors was evaluated by accelerating the corrosion of steel with chloride ions. Specifically, steel disks with a composition similar to commonly used rebars were immersed in simulated concrete pore solutions at pH 10.5 in the presence of chloride ions. The tests were conducted without any corrosion inhibitor (REF) and by adding equimolar amounts of pAB, SA, and CA to the solutions. The occurrence of modifications on the steel surface was evaluated by optical microscopy after immersion in the testing solutions for certain times, such as 20 h, 6 days, 20 days, and 5 months. The micrographs in Figure 2 clearly show that all the three molecules are able to protect the steel surface after treatment in solution for 20 h, whereas the formation of degradation products was observed in the disk used as the reference. By prolonging the corrosion tests up to 6 days, it was found that pAB is the most effective in steel protection. The optical micrographs in Figure 2 show that pAB is able to prevent alterations on the steel surface even after 5 months. On the contrary, the formation of degradation products starts to be detected after 6 days of treatment in the presence of SA and CA (Figure 2). These results suggest that pAB is the most promising among the selected molecules for steel protection.

**Figure 3:**
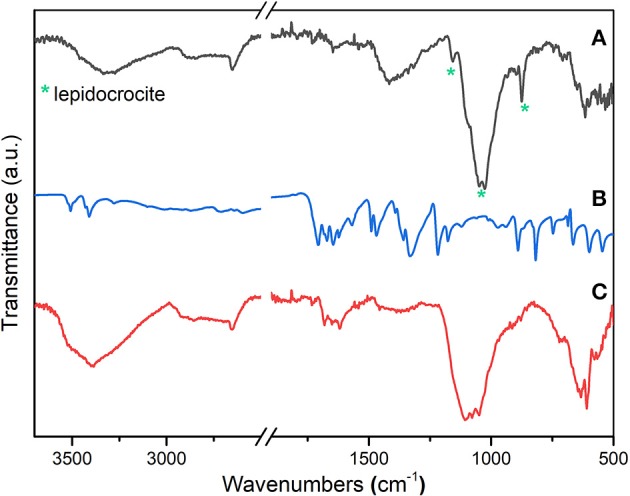
ATR-FTIR spectra of the steel surface after corrosion treatment in simulated concrete pore solutions containing chloride ions. The tests were conducted without inhibitor **(A)** and with *p*-aminobenzoic acid (pAB) **(C)**. The spectrum of the pAB powder **(B)** was reported as a reference.

The surface of the steel disks after the corrosion tests was also investigated by FTIR spectroscopy, with the aim of obtaining information about alteration products and possible interaction with the protective molecules. The results in Figure 3A suggest the formation of a typical iron degradation product, such lepidocrocite (γ-FeOOH) (Veneranda et al., 2018). This could be ascribed to the presence of a broad band at about 1,027 cm^−1^ and to the signals at 1,158 and 871 cm^−1^, which are usually assigned to the lepidocrocite phase. When the treatments are conducted in solutions containing pAB, the FTIR spectrum showed the presence of some weak signals (i.e., in the range from 1,700 to 1,600 cm^−1^), which could be due to inhibitor molecules interacting with the steel surface (Figures 3B,C). This is in agreement with optical micrographs since a chemical interaction between pAB and steel can hinder substrate degradation, preventing surface modifications. No relevant signals were detected in the spectra recorded on the steel surface treated in the presence of SA and CA (data not shown).

These findings clearly show that pAB is significantly more effective than SA and CA in steel protection. Therefore, it was selected as the best candidate to be loaded into silica and LDH containers for the development of stimuli-responsive materials.

### Stimuli-Responsive Systems for a Controlled Release

The pAB inhibitor was used for the functionalization of silica nanoparticles and LDH materials by using two different types of interaction and, consequently, different stimuli-responsive properties. In the case of silica nanoparticles, the inhibitor was chemisorbed through a reaction between the pAB carboxylic group and the amino reactive groups at the silica surface. In the case of LDH, the inhibitor in its anionic form was intercalated into the positively charged lamellar structure.

#### pH-Responsive Materials Based on Silica

##### Functionalization of silica nanoparticles with chemisorbed pAB

Commercial silica nanoparticles SiO_2_-NPs-15, with an average diameter of about 5–15 nm and a high specific surface area of 540 m^2^/g, were first functionalized at their surface with amino reactive groups able to bond pAB protective molecules. According to the literature (Kishor and Ghoshal, 2015), the functionalization with the amino groups (SiO_2_-NPs-15–NH_2_) was carried out by post-grafting the silica surface with organosilanes.

**Figure 4:**
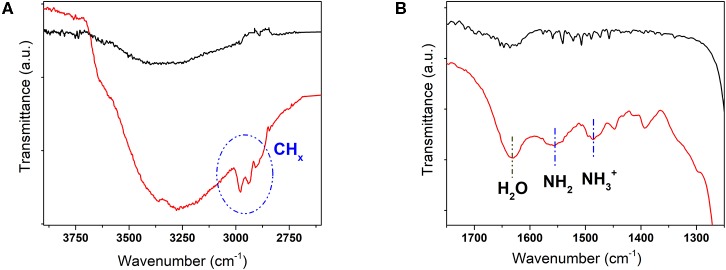
ATR-FTIR spectra of SiO_2_-NPs-15–NH_2_ (*red*) and of commercial SiO_2_-NPs-15 (*black*) in the ranges **(A)** 4,000–2,700 cm^−1^ and **(B)** 1,500–1,200 cm^−1^.

The functionalization success was evaluated by ATR-FTIR spectroscopy. As shown in the spectra of SiO_2_-NPs-15–NH_2_ in Figure 4, the presence of the amino propyl functionalities was confirmed by the absorption band at 1,550 cm^−1^, assigned to NH_2_ bending vibrations, and by the weak band at 1,470 cm^−1^, assigned to the symmetric –NH${}_{3}^{+}$ deformation mode. The presence of the latter suggests that, when the sample is exposed to air, water molecules are weakly bonded to the amine groups, allowing their protonation. The broad bands in the range 2,500–3,500 cm^−1^ associated with the band near 1,630 cm^−1^ are also evidence of the water molecules hydrogen bonded on the silica nanoparticles. The N–H stretching vibration at 3,300 cm^−1^ is more difficult to observe because of its weak dipole moment and the superposition of the absorption of OH groups. In addition, the CH_2_ asymmetric and symmetric stretching modes of the propyl chain are clearly visible at 2,937 cm^−1^ and in the range 2,850–2,920 cm^−1^. The presence of the CH_3_ asymmetric stretching mode at 2,980 cm^−1^ suggests that some ethoxy groups have not been completely hydrolyzed and that, consequently, the chemical-bonded APTES exists in a monodentate and bidentate structure grafted on the silica surface (Pasternack et al., 2008).

**Figure 5:**
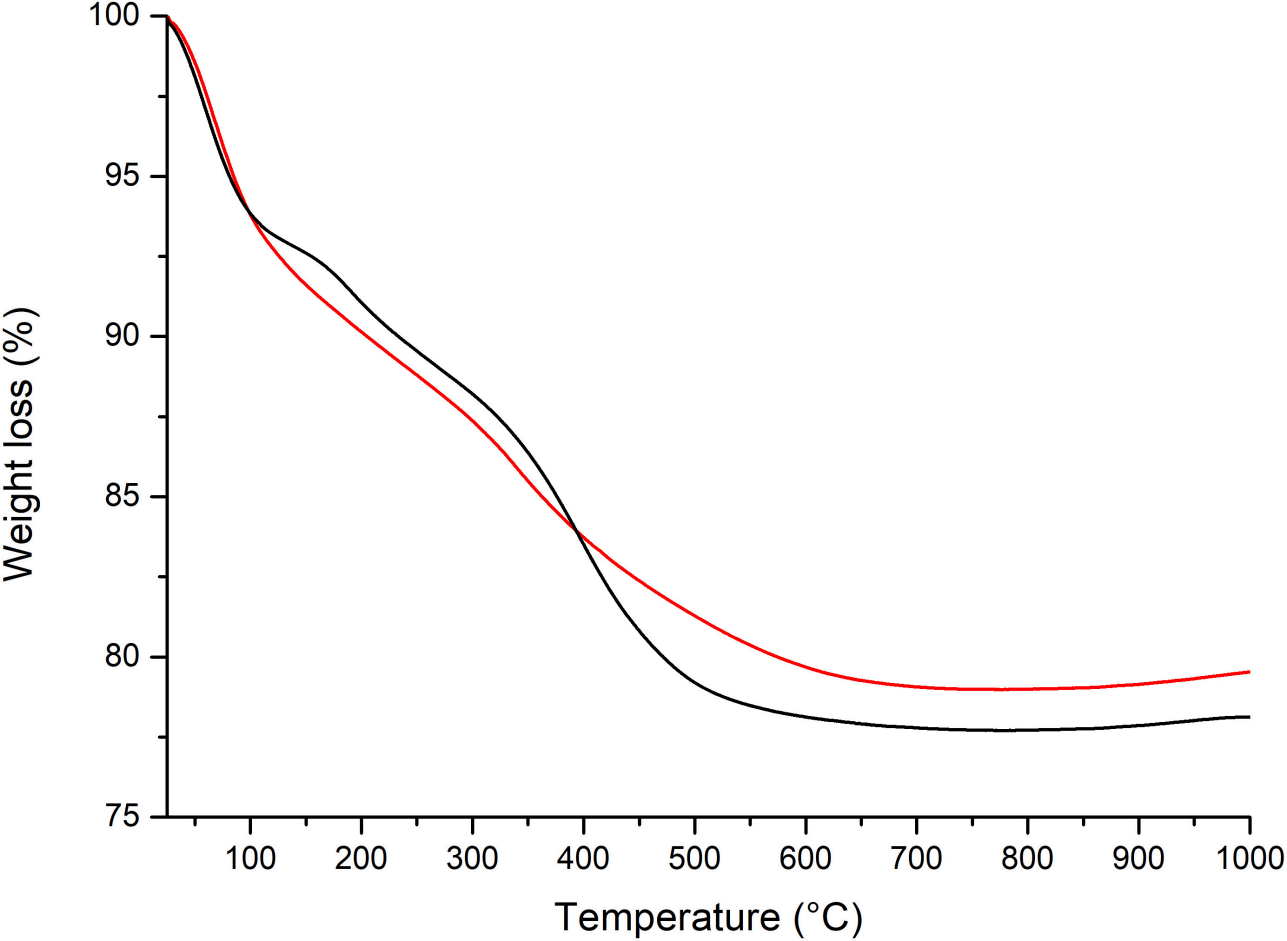
Thermogravimetric (TG) curves in the N_2_ flow of SiO_2_-NPs-15-NH-pAB (*black line*) and SiO_2_-NPs-15–NH_2_ (*red line*).

The organic content grafted on the SiO_2_-NPs-15–NH_2_ surface was evaluated at about 8 wt% (~1.2 × 10^−6^ mol APTES/mg SiO_2_-NPs-15–NH_2_) by thermogravimetric analysis from 300 to 770°C (Qiao et al., 2015). In the case of SiO_2_-NPs-15–NH_2_, the thermogram (reported in Figure 5) shows that the weight loss comes from three contributions (Qiao et al., 2015). The first contribution, in the temperature range of 25–200°C, derives from the elimination of the water molecules adsorbed on the silica surface. At temperatures higher than 200°C, the second weight loss results from the physically adsorbed APTES after ethanol washing. As the boiling point of APTES is 217°C, it is assumed that physically adsorbed APTES is completely desorbed at 300°C. Thus, the third contribution from 300 to 770°C comes from the decomposition of chemically bonded APTES and was used to quantify the grafting.

The amino groups at the silica surface were also qualitatively and quantitatively characterized by the ninhydrin assay. The selective reaction of ninhydrin with primary amine leads to the formation of a Ruhemann's Blue by-product with its characteristic intense absorption band at λ_max_ = 570 nm, thus allowing to assess and quantify the presence of amine groups. The concentration of the amine groups was calculated from the absorption at 570 nm of the SiO_2_-NPs-15–NH_2_ sample by using a calibration curve. The obtained value of 5.8 × 10^−7^ mol APTES/mg SiO_2_-NPs-15–NH_2_ is roughly of the same order of magnitude as that of the TGA. The differences can be explained by some approximations in the calculation from the TGA data.

The functionalization of the silica nanoparticles with the pAB inhibitor was conducted by introducing amino groups at the silica surface and by using them to covalently bond the inhibitor carboxylic moieties (de Oliveira et al., 2016). Therefore, the formation of a pH-sensitive amide bond between the SiO_2_-NPs-15–NH_2_ nanoparticles and pAB was used to provide a tailored release of the corrosion inhibitor in alkaline conditions. The obtained product was labeled as SiO_2_-NPs-15-NH-pAB and was characterized by TGA to evaluate the inhibitor loading. The chemisorbed pAB was estimated at about 2.1 wt% by comparing the weight loss of SiO_2_-NPs-15-NH-pAB and SiO_2_-NPs-15–NH_2_ in the range 300–770°C (Figure 5).

##### pAB release from silica nanoparticles

**Figure 6:**
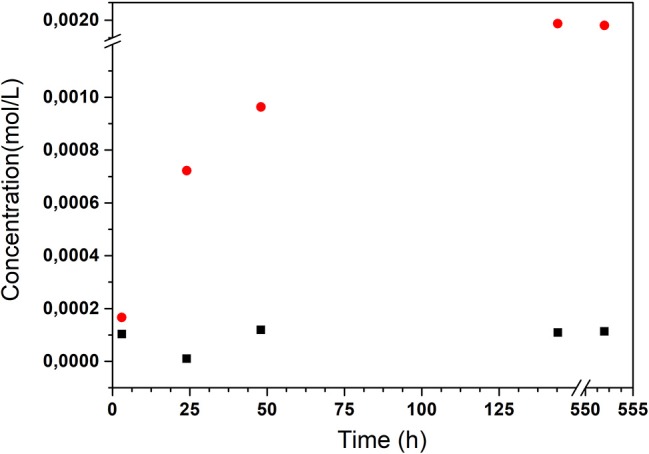
Molar concentrations of *p*-aminobenzoic acid (pAB) released from SiO_2_-NPs-15–NH_2_ after 3, 24, 48, and 144 h of immersion in neutral solution (*black square*) and in alkaline solution, pH 12.65 (*red circle*).

With the aim of investigating the stimuli-responsive properties of the SiO_2_-NPs-15-NH-pAB nanocarrier, this system was studied in neutral environment and alkaline conditions simulating the concrete pore liquid. Defined amounts of SiO_2_-NPs-15-NH-pAB were dispersed in a proper volume of aqueous solution at neutral and alkaline pH, and the pAB release was monitored at different time intervals, up to 552 h (23 days). An alkaline pH of 12.65 was selected since it represents the typical value of the concrete pore solutions. As expected, the experiments show a pH-dependent behavior of the inhibitor release from the nanocarrier. In particular, the pAB concentration in the silica suspension increases with time, from 0 to 144 h, under alkaline pH conditions (pH 12.65), whereas no pAB release is observed at neutral pH. A representative plot of the pAB release trends vs. time at the selected pH conditions is reported in Figure 6. A further increase in the pAB concentration is not detected by prolonging the test up to 552 h (23 days), thus suggesting that the amide hydrolysis is completed after 144 h (6 days) under experimental conditions. It is worth noting that the maximum release of pAB corresponds to about 2 wt% of the SiO_2_-NPs-15-NH-pAB, confirming the inhibitor loading evaluated by TGA.

The obtained results confirm the pH-responsive properties of the developed SiO2-NPs-15-NH-pAB system. It is also worth noting that pAB release continued up to 144 h, due to the gradual hydrolysis of the inhibitor–nanocarrier amide bond, highlighting the potential of this system as an inhibitor reservoir for the long-term protection of steel substrates.

#### Chloride-Responsive Materials Based on Layered Double Hydroxides

##### Intercalation of pAB into layered double hydroxides

**Figure 7:**
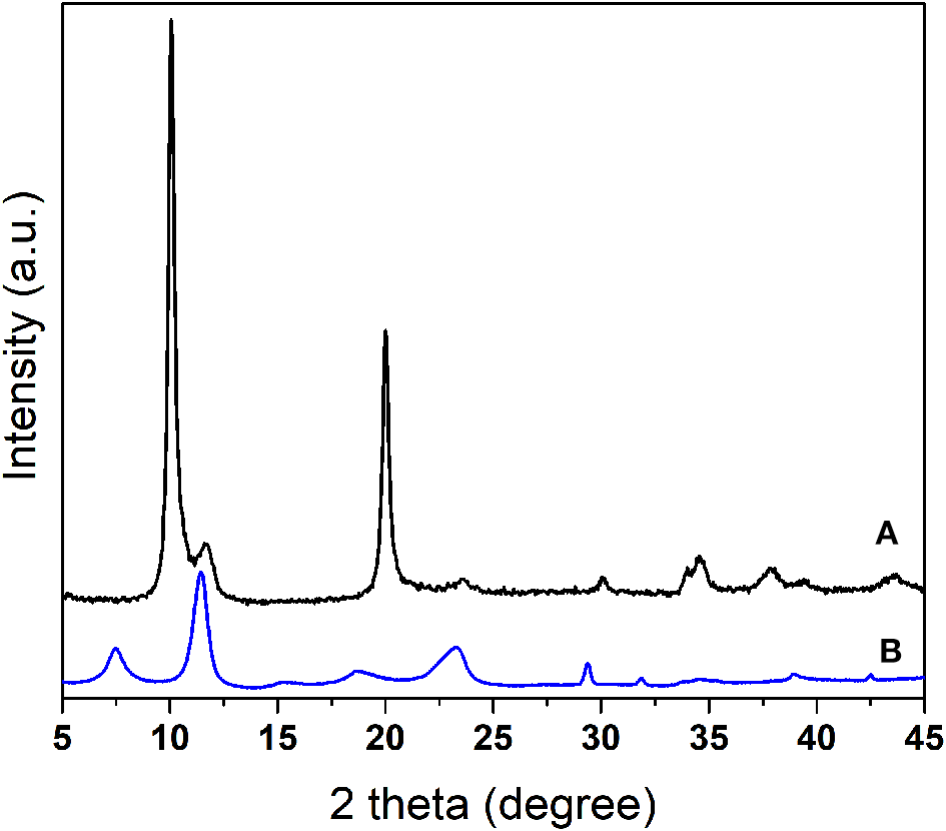
X-ray diffraction patterns of **(A)** LDH-NO_3_ and **(B)** LDH-pAB.

The functionalization of LDH structures with pAB was achieved by using a co-precipitation method for their synthesis. The intercalation of pAB anions into the layered hydroxides was first evaluated by X-ray diffraction measurements. It is well-known that the basal *d*-spacings depend on the size of the intercalated guest anions and can be calculated from the (003) position using Bragg's law (Cavani et al., 1991). The X-ray powder diffraction (XRD) pattern of LDH-pAB is shown in Figure 7, and in order to confirm the intercalation of pAB anions, this profile was compared with LDH-NO_3_. The diffraction pattern of LDH-NO_3_ shows a typical layered structure with high crystallinity and a basal spacing *d*-value of 8.6 Å, comparable to those previously reported in the literature (Grover et al., 2010).

As expected, the pattern of LDH-pAB is rather different compared to LDH-NO_3_. The basal reflections of LDH-pAB are broader and shifted at low angles, thus confirming the expansion of the crystalline structure, with a basal spacing *d*-value of 12.1 Å, due to the intercalation of the inhibitor guest molecules. The expanded interlayer separation is in good agreement with the results reported by Fischer and co-workers for LDHs loaded with pAB molecules (Yang et al., 2016).

**Figure 8:**
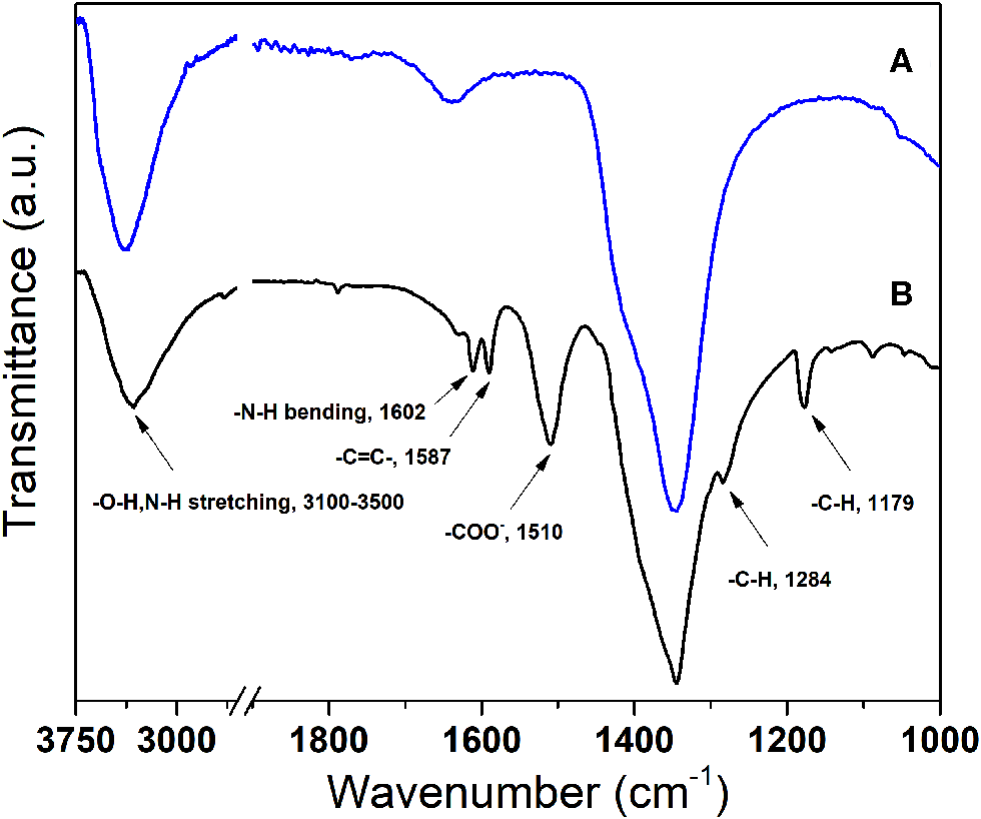
ATR-FTIR spectra of **(A)** LDH-NO_3_ and **(B)** LDH-pAB.

The functional groups in LDHs intercalated with pAB and nitrates were investigated by ATR-FTIR spectroscopy, and their spectra are shown in Figure 8. Complete assignments of pAB bands require the application of an IR method supported by literature data (Samsonowicz et al., 2005). The relatively weak and broad band at around 3,500–3,100 cm^−1^ comes mainly from the O–H groups of the hydroxide layers and from the N–H stretching in the cases of the pAB-modified products (Yang et al., 2016). The peak at around 1,510 cm^−1^ corresponds to the asymmetric stretching vibrations associated with the –COO^−^ of pAB. Furthermore, other characteristic peaks of pAB are observed at around 1,602 cm^−1^ for the N–H bending mode, at around 1,587 cm^−1^ for the aromatic C=C stretching mode, and at around 1,284 and 1,179 cm^−1^ for the C–H bending vibrations of the benzene ring. The presence of these peaks is comparable to literature data for pAB-modified hydrotalcite (Yang et al., 2016) and indicates that pAB has been successfully intercalated into the interlayer space. The FTIR spectrum of LDH-NO_3_ was reported in Figure 8 for comparison. In this case, a peak occurring at 1,650 cm^−1^ can be ascribed to the bending mode of the interlayer water molecules. Additionally, an intense peak at about 1,350 cm^−1^ and a should near 1,400 cm^−1^ correspond to symmetric and asymmetric stretching modes of nitrate. The comparison between the FTIR spectra in Figure 8 confirms that pAB molecules were successfully intercalated into the idrotalcite-like structure even if there was not a complete loss of interlayer nitrate anions and bound water. This is indicated by the presence of a ν3 stretching mode band of NO${}_{3}^{-}$ at around 1,350 cm^−1^ and the O–H stretching band at 1,650 cm^−1^ for interlayer water molecules, although the intensity of both bands was much reduced.

**Figure 9:**
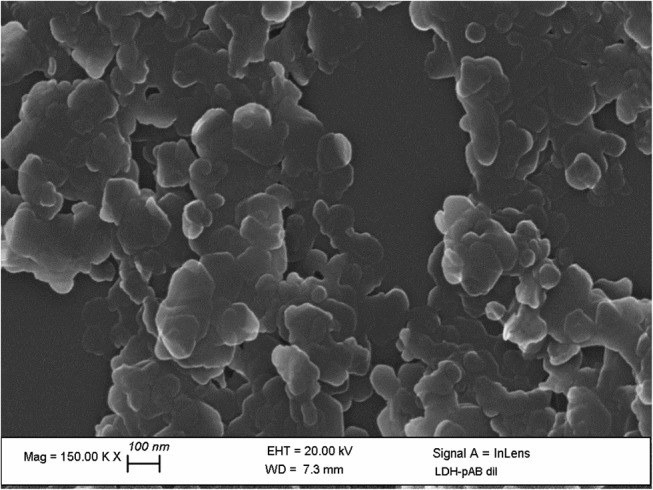
FE-SEM image of LDH-pAB.

The SEM images in Figure 9 show that LDH-pAB particles are composed of round-edged hexagonal particles with a typical plate-like morphology. Probably, the presence of organic molecules in the interlamellar space has an effect on the aggregation between particles. In any case, the sizes of the LDH particles range from about 50 to 150 nm (Wang and Zhang, 2011).

##### pAB release from layered double hydroxides

In the LDHs, the organic anions can be exchanged by aggressive inorganic anions, such as chlorides present in the environment. Therefore, the release of pAB (in anionic form) from LDHs is expected to occur mostly by ion exchange. As such, LDHs play a dual role against chloride-induced corrosion, capturing chlorides as a chloride scavenger and providing corrosion inhibitors in parallel to an internal inhibitor reservoir. Thus, chloride ions can work as a trigger for the release of pAB on demand.

**Figure 10:**
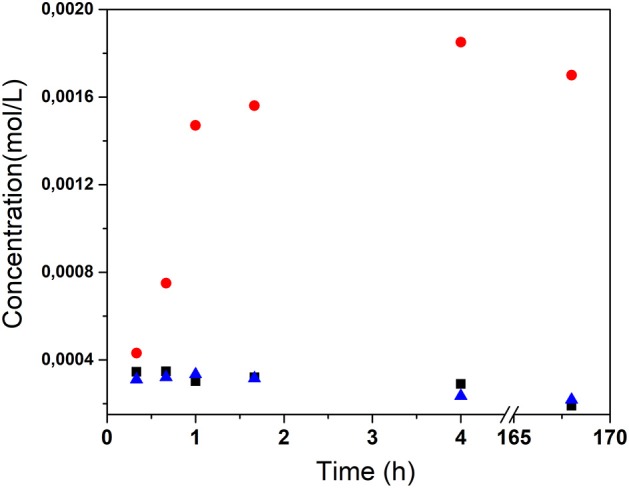
Molar concentrations of the released *p*-aminobenzoic acid (pAB) in aqueous solution at neutral pH and in alkaline solutions in the absence and in the presence of chloride ions.

The stimuli-responsive properties were proven qualitatively and quantitatively by spectrophotometry, adding synthesized LDHs to distilled water, to an alkaline solution at pH 12.65 without and with chloride ions (0.3 M NaCl). The time-dependent release curves of pAB from LDHs are shown in Figure 10. The results clearly show that the concentration of pAB does not increase with time by treatments at neutral and alkaline pH solutions when chloride ions are not present. On the contrary, a selective pAB release by chloride ions was observed, confirming the stimuli-responsive properties of these materials. In this case, the cumulative content of the released inhibitor increases during the first hours of treatment. As shown in Figure 10, most of the protective molecules were released after 4 h, thus leading to an overall released amount of pAB equal to 34.7% in loading.

## Conclusions

This work was aimed at the development of protective materials able to release the loaded inhibitor depending on the chemical environmental conditions. First, the protective efficacy of three corrosion inhibitors containing a carboxylic moiety, namely, pAB, SA, and CA, was compared in simulated concrete pore solutions containing chloride ions. The results showed that, in alkaline chloride solutions, pAB is the most effective protective agent among the three inhibitors considered in this study. When the tests were performed without any inhibitor, the evident formation of degradation products was noted after only 20 h of treatment, and the surface characterization by FTIR spectroscopy suggested mainly the presence of lepidocrocite among the iron corrosion products. When the tests were conducted in the presence of SA and CA inhibitors, corrosion processes on the steel surface were delayed and observed after only 6 days. It is surprising that, even after 5 months of corrosion treatments in alkaline chloride solutions containing pAB, the steel surface remains unchanged.

Based on the results from the corrosion tests, pAB was selected and used for the functionalization of silica nanoparticles and LDHs. The idea was to confine the protective agents into nanocarriers that can gradually release them on demand. Specifically, pAB was chemisorbed through its carboxylic moiety on silica nanoparticles with surface amino groups. Our findings confirm that pAB chemisorbed *via* an amide bond is gradually released at alkaline pH. It is worth noting that pAB release continued up to 144 h, highlighting the potential of this system as an inhibitor reservoir for the long-term protection of steel substrates.

Furthermore, pAB was intercalated in its anionic form into the LDH structure. In this case, the release mechanism is completely different since it is induced by the presence of chloride ions and occurs by an anionic exchange reaction. Thus, these materials play a dual role by acting as an inhibitor reservoir and by capturing chlorides. A gradual release of pAB was also observed for LDH-based materials, but, in this case, the release continued up to 4 h. However, an important advantage of LDH-pAB is the high inhibitor loading and release which corresponds to 34.7 wt%.

In summary, it is possible to create a reservoir of corrosion inhibitor which is released on demand based on the chemical environment. By selecting an appropriate nanocarrier, it is possible to tune the inhibitor release, which is triggered by alkaline environments for silica-based materials and by chloride ions for LDH-based materials. Our findings confirm that, by using different nanocarriers, it is possible to obtain stimuli-responsive materials with a complementary protective action. The gradual and tailored release of the pAB inhibitor is attractive since this can provide long-term protection of steel. These materials could be applied on the concrete surface or directly dispersed into the concrete matrix during the preparation, thus opening the way for innovative solutions in the preservation of concrete cultural heritage.

## Data Availability Statement

The datasets generated for this study are available on request to the corresponding author.

## Author Contributions

CG and EM contributed to the design of the work and to the material synthesis. MS and MP contributed to the corrosion tests and spectroscopic analysis. CR and LT contributed to the optical and SEM analysis. LL contributed to the TGA analysis. GI contributed to the revision and commented on the manuscript. GD designed the research and prepared the manuscript.

## Conflict of Interest

The authors declare that the research was conducted in the absence of any commercial or financial relationships that could be construed as a potential conflict of interest.
